# In silico modeling for tumor growth visualization

**DOI:** 10.1186/s12918-016-0318-8

**Published:** 2016-08-08

**Authors:** Fleur Jeanquartier, Claire Jean-Quartier, David Cemernek, Andreas Holzinger

**Affiliations:** 1 Holzinger Group, Research Unit HCI-KDD, Institute for Medical Informatics, Statistics and Documentation, Medical University Graz, Auenbruggerplatz 2/V, 8036 AT Graz, Austria; 2 Institute of Information Systems and Computer Media, Graz University of Technology, Inffeldgasse 16c, Graz, 8010 AT Austria

**Keywords:** Cancer, Tumor growth, In silico, In silico medicine, Visualization, Visual analysis, Computational biology, Cellular Potts model, Glazier and Graner model, Cell proliferation

## Abstract

**Background:**

Cancer is a complex disease. Fundamental cellular based studies as well as modeling provides insight into cancer biology and strategies to treatment of the disease. In silico models complement in vivo models. Research on tumor growth involves a plethora of models each emphasizing isolated aspects of benign and malignant neoplasms. Biologists and clinical scientists are often overwhelmed by the mathematical background knowledge necessary to grasp and to apply a model to their own research.

**Results:**

We aim to provide a comprehensive and expandable simulation tool to visualizing tumor growth. This novel Web-based application offers the advantage of a user-friendly graphical interface with several manipulable input variables to correlate different aspects of tumor growth. By refining model parameters we highlight the significance of heterogeneous intercellular interactions on tumor progression. Within this paper we present the implementation of the Cellular Potts Model graphically presented through Cytoscape.js within a Web application. The tool is available under the MIT license at https://github.com/davcem/cpm-cytoscapeand http://styx.cgv.tugraz.at:8080/cpm-cytoscape/.

**Conclusion:**

In-silico methods overcome the lack of wet experimental possibilities and as dry method succeed in terms of reduction, refinement and replacement of animal experimentation, also known as the 3R principles. Our visualization approach to simulation allows for more flexible usage and easy extension to facilitate understanding and gain novel insight. We believe that biomedical research in general and research on tumor growth in particular will benefit from the systems biology perspective.

## Background

Around 13 % of all deaths worldwide are due to cancer [1]. Cancer depicts a group of diseases which refer to abnormal new growth of cells which can spread and invade different areal parts throughout the body [2]. A tumor is most commonly described as an abnormal growth of clustered cells which can be either benign (well-structured and non-harmful) or malignant (cancerous) [3]. Treatment against cancer directly relates to the growth-behaviour rendering the onset of therapy critical for its outcome. As a matter of fact, oncology is primarily based on prediction aspects [4]. In this regard, we focus on the assessment and prediction of tumor growth. The growth of tumors depends on their supply of oxygen, nutrients as well as survival factors and is influenced by growth factors as well as its local surroundings [5]. Characteristics are individually based on the different types of tumors [6]. The mathematical basis for tumor growth has been described in the mid of the last century not to be exclusively exponential but to be following a continuous deceleration as presented by the Gompertz function [7, 8]. Modern approaches, for example, take the heterogeneous subclonal mixtures [9] of tumor cells into account or even its interdependency to cellular motility [10]. Our model includes basic ideas of tumor growth, set for further enhancement through multiple expansion possibilities. We apply in-silico modeling of tumor-growth as a primary tool, and further advance it to a novel Web-based simulation, evenhandedly available for biomedical scientists and clinicians with a focus on feature visualization. Features are key to learning and understanding. Thus, features are of enormous importance for knowledge discovery.

### Computational modeling in biomedical science

These days, biomedical science heavily relies on computerized support for analyzing big data, quantifying dynamic and multiscale events, or likewise for simulating complex models. Computational models have been applied for intra- and inter-cellular, tissue- and organ-specific aspects [11]. Additionally, there is the ongoing project of creating a virtual physiological human [12] in order to support clinical decision-making. The project includes multi-level modeling of a wide range of information dealing with patient-specific signaling and genetic data up to whole-organ physiological mechanisms.

There are two main advantages of the bioinformatic approach in computational modeling of disease. First, simulations can be used for predictions in regard to the basic idea of alternative testing methods in addition to or instead of laborious experimentation. Alternative testing methods comprise the categories of replacement, reduction as well as refinement of in-vivo experimentation, that are summed up by the 3R principle [13, 14]. Thereby, in-silico methods are applied to in-vivo and in-vitro extrapolation [15, 16]. Secondly, prediction models overcome the lack of experimental methods for insufficient or nonexistent early screening tests. In general, models can be used to gain insight into complex biological systems and may address the gaps in literature as well as form the foundation for future research [17–19]. Simplification and approximation of the numerous detailed information gained from biomedical science offers the possibility to patient-personalized prediction, avoids hard-to-measure variables or compensates non-measurable factors [20]. Still, models are, so far, inflexible to simple extensions or even rescaling. Furthermore, we have to overcome the conflict between complexity and oversimplification. For instance, global mapping of cell community is computationally too laborious while the averaged approach lacks detailed description of molecular variables [21]. Still, in silico modeling and other computational techniques help answering key questions in cancer research [22–25].

We emphasize the approach of computational modeling of biological systems and developing computational modeling tools for simulation and reproducibility of experiments in biologic research. Fisher et al. [26] coin the term Executable Biology which highlights the difference between mathematical and computational bio-models in regard to their representation. Executable Biology describes computational algorithms in support to reproducible results in biomedical research as well as efficient simulation and analysis of biological systems. In this regard, Executable Biology is recommended to be integrated as standard method into bio-science.

Regarding the dynamics of tumor growth, computational models for various types of tumors exist, from animal models and the human body, dealing with the individual stages of tumor development [27]. In silico cancer modeling provides significant opportunities, however, Edelman et al. [28] argue that it is yet in its infancy.

Understanding the tumor heterogeneity with respect to personalized cancer treatment represents the ultimate goal of computational tumor-growth modeling. For that matter, multiple groups of scientist have to work together, accentuating the need for interchangeable infrastructure of linking big data and adoptable specialized models [29].

### Mathematical modeling of tumor growth

Tumor growth kinetics follow relatively simple laws that can be mathematically described [30]. Such mathematical models could forecast individual phases of tumor growth [31]. In general, there are basic modeling approaches of cancer kinetics [28], that include exponential growth, the Gompertz model [32], metabolic models [33], the so-called universal model [34] and hybrid models [35]. Various mathematical models have been developed for the description and prediction of tumor growth. Each model, available so far, is optimized for specific scales of time and size plus certain aspects of metabolism or interactions [28, 35]. In regard to different biological scales, Deisboeck et al. [36] discuss innovative multi-scale cancer modeling approaches, ranging from atomic and molecular up to macroscopic scale. However, there is no universal law yet. Simple models have prediction rates less than 70 %, while some models used for specialized simulations achieve ≥80 *%* prediction rates [30]. Cancer models can be categorized based on their basic mechanisms to calculate tumor growth, but several additional factors have to be considered. Tumors originate from differentiating cells exhibiting the behavior of excessive proliferation up to migration [20, 37]. Tumors can be either dormant or growing [38, 39]. After reaching a critical mass, primary tumor growth stops and migration through metastasis will occur. From a biological perspective, tumor growth also depends on the underlying network structure [40–42].

### Cellular Potts modeling of tumor-growth

The Cellular Potts model (CPM) poses a most widely used example of agent-based models which are feasible for research regarding cell-based phenomena and, therefore, are favorable for cancer research [43, 44]. The CPM was first presented by Graner and Glazier [45, 46]. The CPM or also named Glazier-Graner-Hogeweg (GGH) model is based on individual cells in contrast to continuum models which summarize cell populations to tissues and continuous materials [47, 48]. It represents a modeling approach on tissue level with the main focus on intracellular and intercellular events as well as the cellular microenvironment. It has been implemented for tumor progression and invasion before [43]. The model includes single-cell characteristics of cellular geometry and interactions, rendering the simulation more efficient for questions on a detailed level than for a general overview. Glazier and Graner’s model was originally developed for simulating the rearrangement of individual cells and cell sorting [46]. They upgraded the model to a compartmental view of cellular subelements. In principle, various cells are described as objects covering multiple shifting nodes on a 2D or 3D lattice while moving and changing their size. Thereby, CPM simulations support studies on type-specific cellular morphology and interaction [49]. The model describes different cell states and allows for additional parameters such as cell division and migration [50] as well as chemical diffusion and the extracellular matrix (ECM) [51]. Graner et al. [45] showed that differential cell adhesion and chemotaxis can be controlled through CPM, while the model is robust in regard to certain parameter choices. Glazier et al. [47] revise several development steps of the CPM and Szabo et al. [43] summarize the usefulness of CPM for simulating multi-cellular processes related to cancer. Boas et al. [52] recently conducted a global sensitivity analysis of the CPM, taking model extensions for angiogenesis into account, and showed that introducing a dynamic parameter for chemoattraction has the highest impact, being followed by the diffusion coefficient and cell-cell adhesion.

CPM has been used in a wide range of applications and there are extensions in terms of kinetics also referred to as extended CPM as well as hybrid CPM models [49]. The background of CPM modeling on cell sorting for various cell-types has been successfully used for the simulation of benign tumor growth [53] and cancer invasion [54]. Moreover, multiscale-models based on CPM have been implemented for various cancer-related studies [43, 51, 55–60].

### Visualization for computational modeling

Visualization supports the understanding of biological data and provides insight into biological systems [61]. Visualization and computation mutually contribute to the sense-making process of biomedical analysts [62]. It is advised to provide integrated frameworks for biological studies. Graphical representations used for biological data visualization need to be adjusted to an appropriate level of detail. Graphs, in which each node represents a biological object and each edge a relation between these nodes, are often found in visualizations of biological data. While it has been primarily used for large interaction networks so far, graph visualization offers several user-friendly layout algorithms and is applicable for a wide are of application areas, ranging from social networks, finance to biology [61, 63]. Our recent study [64] on integrated visualization of biological networks highlights current possibilities for using Web technologies to support analysts in exploring biological relations.

The field of computational cancer biology lacks visualization types apart from network visualization. The “cBioPortal” with its focus on cancer genomics offers interactive visualization of pathway networks, mutations in protein domains, statistical information and trends on gene sets and clinical patient data of 10 published cancer studies [65]. Besides, there are only a few attempts on integrating visualization in computational modeling tools for cancer biology. Simulation results of a multiscale model for glioma growth have been visualized by the use of the software SciRun [66]. Specific cell growth processes can be simulated and visualized with the tool CellSys [67]. CompuCell3D [68] and the Tissue Simulation Toolkit [69] are exemplary frameworks for testing and extending computational models, integrating visualization features on cell interactions for simulation and analysis. Last but not least, there have been efforts in developing a virtual biobank [70] and a cancer modeling community [36] to exchange data and to facilitate visualization integration.

Though computational modeling has become a feasible tool for tumor growth research, simulation tools are rare. There is a step by step tutorial available how to simulate collective cell behavior based on Cellular Potts modeling [71]. CompuCell3D is one of these tools which has been used for in silico modeling of cellular and multi-cellular behaviors [68]. The latter research group introduces a tutorial for building cell-based simulations for visualizing tumor growth by making use of an open source library for simulating the CPM, written in C++. Though providing step-by-step instructions, basic knowledge of the use of the terminal and a C++ compiler are required. This technical know-how is often a limitation to clinicians and researchers in biomedical sciences. Moreover, they do not describe how to create iterative computations and how to differentiate between cell-types.

However, despite the availability of many different tumor growth models on the one hand and many Web-based visualization libraries on the other hand, adequate and usable simulation tools are still rare. To our knowledge, there have been no efforts in creating easy to use, Web-based computational cancer modeling tools that integrate visualization features. Our main idea is creating usable and extendable implementations of tumor models to foster ease of use of simulations and support knowledge discovery.

## Methods

### Mathematical basis of tumor growth

In general, tumor growth is mathematically summarized by the Gompertz function [7, 8, 32, 43]:

$$ \frac{V_{t}}{V_{0}} = e^{\frac{a}{x*\left(1 - e^{- at}\right)}} $$

with tumor size at variable time *V*_*t*_ and the initial tumor size *V*_0_, *a* and *b* being tumor-type characteristic constants, for cell clone division [7, 8]. In detail, we choose to describe tumor growth using the CPM by GGH where the probability for a spin copy and therefore cell proliferation is expressed as:

1 $$ p\left(\sigma_{i,j} \rightarrow \sigma_{i',j'}\right) = \left\{\begin{array}{ll} e^{\frac{-\Delta H}{T}} & \text{if } \Delta H > 0; \\ 1 & \text{if } \Delta H \le 0; \end{array}\right.  $$

The CPM is a time-discrete markov chain and its transitions $\sigma _{i,j} \rightarrow \sigma _{i',j'}\phantom {\dot {i}\!}$ are calculated by a Hamiltonian (or energy) function *ΔH*, a sum of several terms [46, 47]. We further describe details on its implementation within the next subsection.

### Implementation of the CPM

The Potts model is based on the differential adhesion hypothesis which states that motile cells rearrange themselves according to the lowest energy configuration along the potential energy landscape [46, 72]. Within the CPM by GGH, cells are assigned certain spin states. Cells are build up by multiple cellular bricks, likewise termed (cellular) lattice nodes, sites or points. A multi-scale growth is accomplished through surface adhesion and space competition of *cellular bricks* scattered through the discrete lattice. *Cellular bricks* are associated with spins at lattice sites. Spins can be flipped between spin states allocating a celluar brick to another cell. These spin-copy attempts are calculated through Monte Carlo Steps (MCS). MCS are the mathematical basis for the probability simulation. The key parts of the computation are the Hamiltonian function *ΔH*, also referred to as configuration energy [47], shown in Eq. 2, and the temperature *T* shown in Eq. 3.

2 $$ H = J \sum\limits_{i,j} \left(1- \delta_{\sigma_{i,j} \sigma_{i',j'}}\right)  $$

If *ΔH*<0 the new spin state is always accepted because the system’s energy will be decreased. If *ΔH*≥0 the new spin state is accepted with a certain probability. While the cell is growing its target volume increases too. A *cell* in the CPM is the set of all *cellular bricks* with the same cell-index. Each cell relates to a certain cell-type. The cell-types are defined by the set *τ*.

*ΔH* constitutes the energy of interactions between cellular bricks *i* with the neighbour *j*. The discrete version of the Kronecker delta *δ*=1 if two neighbouring bricks are from the same cell, otherwise *δ*=0.

A cell will reach a critical point for division upon minimum *ΔH*. Each cellular brick is assigned a *σ*_*i*,*j*_ with type-dependent interaction energies, the spin-spin coupling energy constants *J*(*σ*_*i*,*j*_) to neighbouring cells. *J* effects a cell to be inclined to comprise a formation of connected cellular bricks over loose entities.

*MCS* is a series of *n* spin-copy attempts for a lattice consisting of *n* lattice sites. Each MCS step resembles the rearrangement of cells and, therefore, the time. The calculation shown beneath includes the temperature *T* which resembles a cellular motility factor [47]. The MCS calculates a change in configuration of *H*_0_ to *H*_1_ for:

3 $$ \Delta H = H_{1} - H_{0} \le 0 \text{~or otherwise~} p = e^{\frac{-\Delta H}{T}}  $$

The CPM Hamiltonian *H* is the sum of a series of terms that are related to different cell attributes such as interaction energy as well as volume. Extended versions exist that include other addends [49]. The original CPM includes a second term next to the first term of all surface energies *J*. *H* also includes a *λ* as cellular constraint as function of elasticity, shown in Eq. 4.

4 $$ H = J \sum\limits_{i,j} \left(1- \delta_{\sigma_{i,j} \sigma_{i',j'}}\right) + \lambda \sum\limits_{\sigma} (v(\sigma) - V_{t}(\sigma))^{2}  $$

In more detail, *H* includes the number of lattice sites *v*(*σ*) in a given domain with the spin *σ*, and the target number *V*_*t*_(*σ*) within that domain. The second term confines a cell’s volume *v* to the range of a specific target volume *V*, while the variable *σi*^′^*j*^′^ sums up the number of neighbours. We focus on a schematic two-dimensional cellular grid. A cell’s volume *v* and target volume *V*_*t*_ is thereby reduced to area *a* and *A*_*t*_.

### Web-based model implementation

The implementation for the purpose of visual analysis of tumor growth includes:

- CPM implementation, based on Glazier et al. 1993 [46]
- Servlet for client-server communication
- Network visualization based on Cytoscape.js [73]
- Line chart visualization based on Flot [78]
- HTML5 frontend
- Tests
- Documentation

**Fig. 1:**
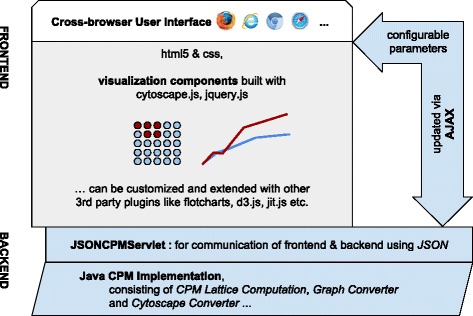
Architectural Representation of cpm-cytoscape: The architecture is composed of two distinct layers: The *frontend* layer contains a Cross-browser web presentation layer. It contains the customizable visualization components as well as a dynamic user interface for interacting with the CPM implementation via AJAX (with GET/POST). At the *backend* layer the JSONCPMServlet serves as interface for the actual CPM Lattice computation in the backend

The CPM is implemented as server side backend. Thereupon a cross-browser user interface integrates client side visualization libraries for multiple visualization outputs (Fig. 1).

The presented tool *cpm-cytoscape* offers an HTML5 based graphical user interface that makes use of JavaScript (JS) libraries, first and foremost *Cytoscape.js*. Below the frontend, the backend is implemented in JAVA and information between frontend and backend is exchanged in JavaScript Object Notation (JSON), a common data exchange format that is used by Cytoscape.js. The JSON data holds a reference for the output container as well as several elements. The elements further contain child elements such as the complete set of edges and nodes, while each node again contains data about id, position, color, neighbour, parent, selection and other parameters. Moreover, the JSON structure includes information about the graph’s layout and style parameters. By making use of a Java implementation of the CPM computation, a set of Java Servlets are requested asynchronously and delivering the data needed both for the computation in the backend and for the visualization rendering in the frontend.

### Visualization

We developed an HTML5 frontend that can be easily adjusted by means of modern web design via editing markup, JS and presentation stylesheets. The frontend can further be extended by integrating additional control elements as well as by making use of additional JS-based visualization libraries. For the visualization we searched for a library capable of rendering nodes along a lattice, and we found Cytoscape.js to be the graph visualization library of our choice. We use visualization libraries to create and update the visualization during a simulation run. The rendering method requests the *JSONCPMServlet*, a Java servlet that delivers data needed for the frontend rendering. Therefore, the JSONCPMServlet first receives JSON data, parses it, maps it and sends it back as JSON, that is then used for the graph rendering. For now, the frontend rendering parts include a graph visualization and a simple line chart. We use *Cytoscape.js* to plot the lattice-based graph visualization as well as *Flot*, a *Jquery.js* extension, to draw simple line charts.

### Usage of cpm-cytoscape

Based on a study on a brain cancer type modelled by CPM [51] and our ongoing work on tumor growth profiles for simulation [74] we introduce the tool through a short tutorial at https://github.com/davcem/cpm-cytoscape. We encourage readers to use GitHub for having a closer look at our implementation, explore its features and suggest enhancements as well as participate in the development. Design and implementation of the presented tool took place in an iterative manner. Informal validations have been conducted by several discussions with a domain expert. The basic idea up to the model’s implementation and the tool’s user interface have been co-designed and reviewed by a domain-expert.

## Results

We present a new 2D visualization approach for a dynamic cellular model simulation that accounts for lattice size, cell size, environment parameters and interactions between cells. The tool developed and used for the simulations has been published in the GitHub repository, saved as cpm-cytoscape. It can be obtained via the url address: https://github.com/davcem/cpm-cytoscape. Further, we provide a demo version that is online available on: http://styx.cgv.tugraz.at:8080/cpm-cytoscape/.

We created the tool to allow for easy manipulation by its user. The upper region offers a number of variables which can be set by the user in order to discriminate and process various experiments. The CPM is computed solely in the Java backend, while initialization parameters can be adjusted in the frontend and are communicated by requesting the servlet. By varying several parameters the user is allowed to simulate a wide range of conditions. These parameters are the lattice’s size (*x,y*), the count of monte carlo steps, its’ substeps, *max σ*, the *matrix density*, *interaction parameters* as well as the *temperature*. The Java packages consist of the implementation of the CPM itself, a graph converter to convert the CPM lattice into a graph structure, a more specific cytoscape converter to represent the graph enrichment needed for the visualization library as well as the servlet to provide the communication interface between backend and frontend via JSON.

Individual cells are visualized as group of nodes, we refer to as *cellular bricks*, on a grid. Cytoscape.js provides a grid layout rendering algorithm that arranges the nodes in a square grid whereby the circular nodes represent subcompartments of cells. We differentiate between *light* cells that represent normal cells, *dark* cells that represent mutated cells and the ECM that surrounds cells. The ECM is represented as grey nodes. The other nodes with *σ*≥1 are represented by the colored, either dark or light nodes. For now, we only differentiate between a light and a dark cell-type. Nodes which are not indexed as light or dark cells are attributed to the ECM. They resemble the cellular surroundings without peculiar growth variables.

The *growth rate* can be visualized as line chart for *σ*=2 by using the button “show line chart”. The line chart shows the amount of computation steps on the x-axis and the amount of cellular bricks on the y-axis. Experimental data can be exported as spreadsheet in the format of comma-separated values. This option offers the possibility of making the data available offline for further analysis.

**Fig. 2:**
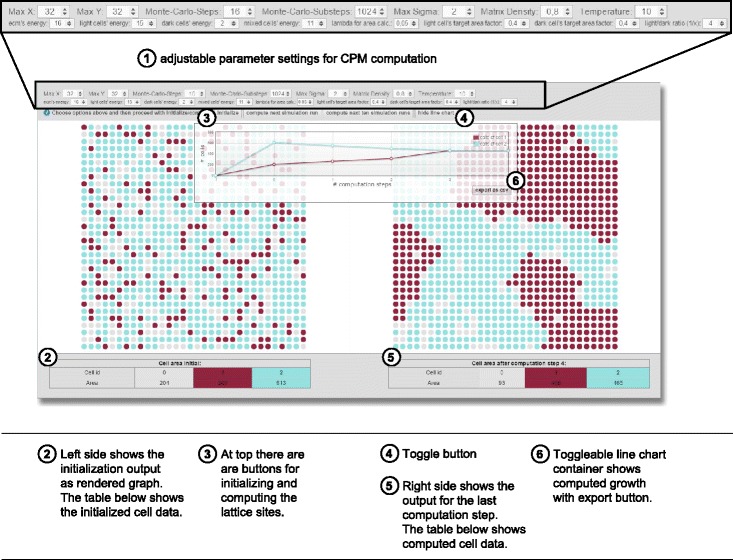
Overview of the tool’s user interface: ① At top there are adjustable parameter settings for CPM computation and ③ buttons for initializing and computing the lattice sites. ② The left side shows the initialization output as rendered graph with grey nodes representing parts of ECM, while colored nodes representing cellular bricks corresponding either to light blue colored (normal/healthy) or dark red colored (tumor/mutated) cells. The table below shows information about the initialized cell data. ⑤ The right side shows the output for the last computation step, while the table below contains computed cell data. ④ A toggle buttons controls the ⑥ lightbox in the middle that provides line chart visualization and export

**Initialization and lattice settings:** The lattice is created on the left side of the browser window by pressing the button *initialize* (Fig. 2). Thereby, the size and likewise the number of nodes is determined by the input of variables *x* and *y*. This allows to adjust the experimental area. Nodes are indexed randomly to *light* and *dark* cells or *ECM* according to the input of the number of cellular clusters *σ*, *matrix density* and the *light/dark ratio*. After initializing a random graph according to the user interface’s settings the computation possibilities with the button “compute next simulation run” and “compute next two simulation runs” are enabled (Fig. 2).

**Fig. 3:**
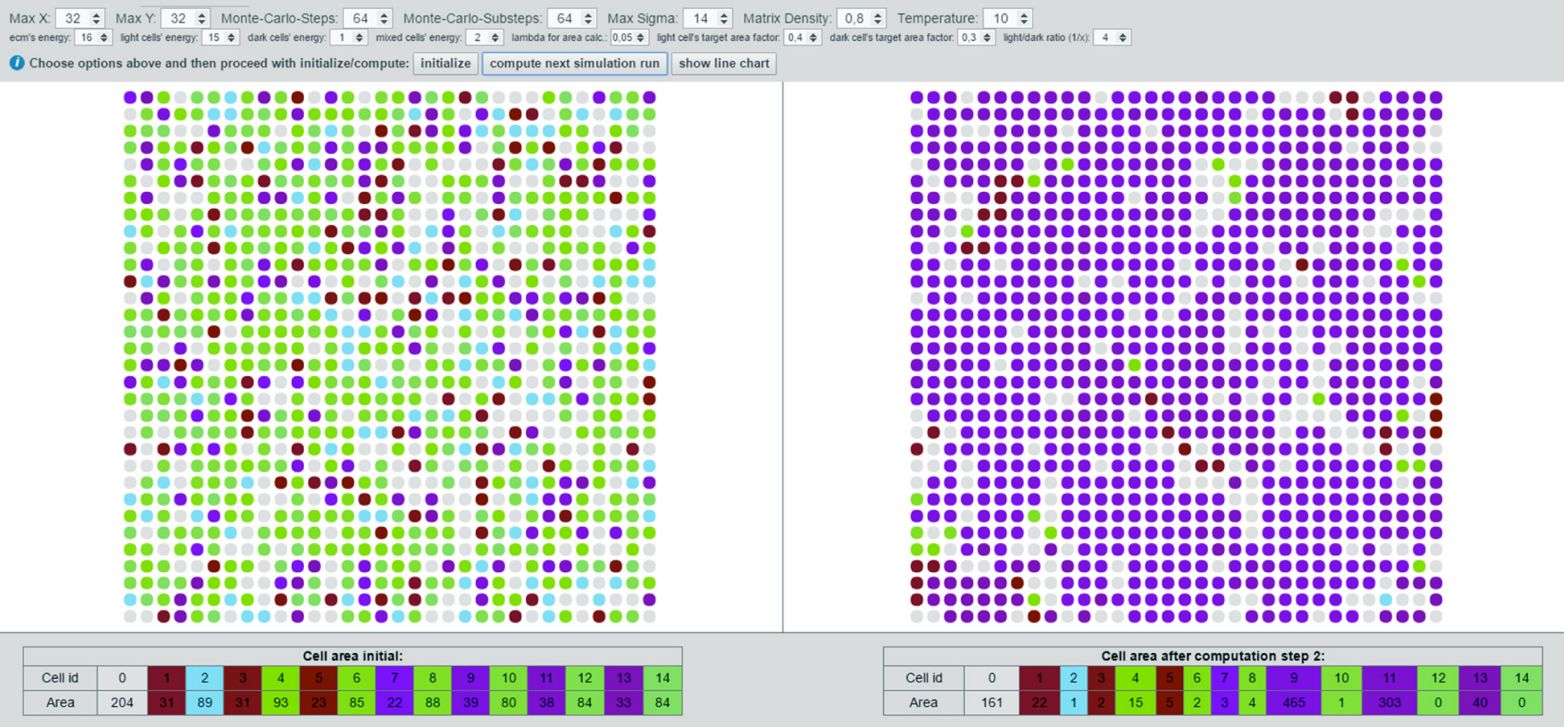
Screenshot of a graph rendering with *σ*_*max*_=14: The graph consists of 14 distinct cells (also denoted to as cellular clusters). Each cell is represented by a certain amount of nodes that we call cellular bricks. Cellular bricks with dark red or purple color tones correspond to the dark (tumor/mutated) cells, while nodes in a light shade of blue to green are referencing the so called light (normal/healthy) cells. The amount of these are listed in the table below the graph visualization. For this example, the cell with *i* *d*=9, represented by the purple nodes, consists of 39 cellular bricks at the initialization phase. After 2 computation steps, we see at the right side, that the cell with *i* *d*=9 has grown and now holds 465 cellular bricks. Grey nodes represent the ECM

Our implementation of the CPM currently consists of *maxSigma* cells relating to 3 different cell-types, while *σ*=0 attributes to the ECM, the odd numbers refer to dark cells and the even numbers to light ones. Therefore, by making use of the **max***σ* parameter one can also define more than two different cells, also referred to as cellular clusters. *Max σ* defines the quantity of individual cell components or respectively cellular clusters. If max *σ* is set to 2 we use the color lightblue for light (normal/healthy) cellular bricks and darkred for the dark (tumor/mutated) ones (Fig. 2). If max *σ* is set to >2 we use a colorscheme for coding dark and light cell nodes slightly differently to better distinguish between different *σ*, shown in Fig. 3. The factor *σ* can be redefined to resemble the number of cell-types. The cell-types are represented by *τ*, in some papers also referred to as cell or medium. We currently distinguish between three cell-types, namely ecm, light and dark cells as denoted in the original paper by Graner et al. [46]. A cell-type is referred to as *τ*_*i*_, while *τ*={0,1,2} with *τ*_*i*=0_=*ECM*, *τ*_*i*=1_=*dark*, *τ*_*i*=2_=*light*.

The *matrix density* defines the number of cellular bricks indexed as light or dark cells in relation to the given number of nodes. Setting *matrix density* =1 uses all lattice sites for cellular bricks. Setting *matrix density* =0 represents a lattice site filled only with ECM.

The parameters *MCS* and #*substeps* represent units of time, while a substep is related to a random copy attempt. We implemented the number of *MCS* and *substeps* as variables and allow the parameters to be defined and adjusted by the user. Each *MCS* is divided into a specified amount of *substeps* for simulating different time settings.

**Fig. 4:**
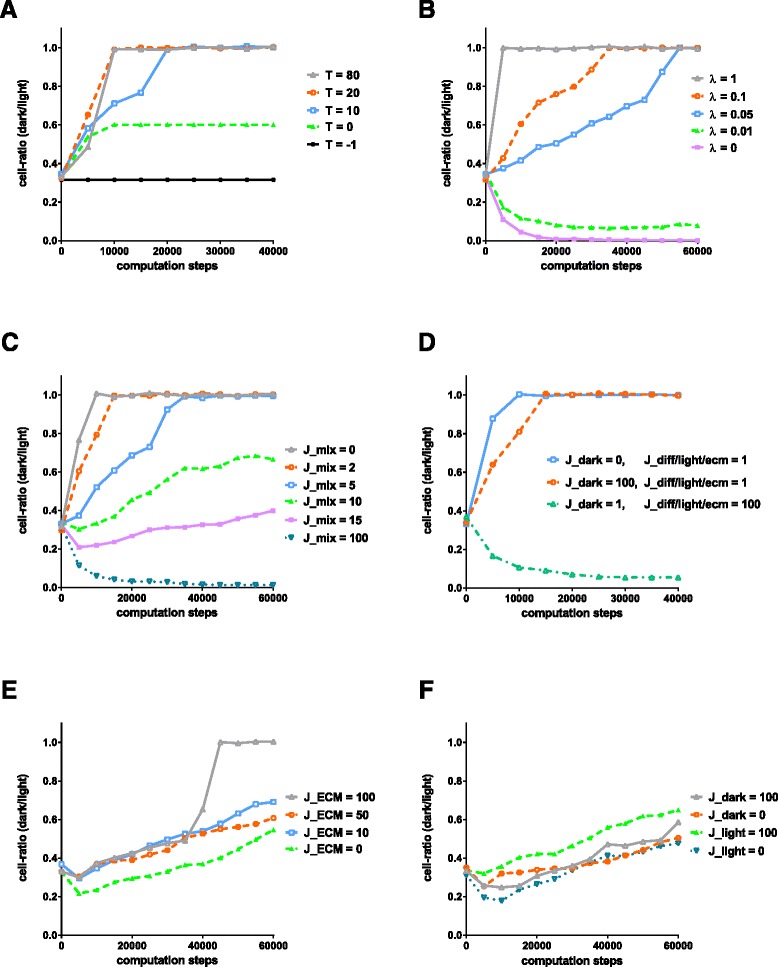
Cell growth in relation to varying parameters: line chart showing representative ratios between numbers of dark and light cellular bricks over computed steps. Comparison of varying parameters, for temperature *T*=80,20,10,0,−1 (panel A), *λ*=1,0.1,0.05,0.01,0 (panel B), *J*_*mix*_=0,2,5,10,15,100 (panel C), comparison of various *Js* as indicated for *J*_*dark*_, *J*_*light*_, *J*_*mix*_, *J*_*ecm*_ (panel D), *J*_*ecm*_=100,50,10,0 (panel E), *J*_*dark*_ and *J*_*light*_ each 0 or 100 (panel F). Adjusted to default settings of *n* *o* *d* *e* *s*=32∗32, *m* *c* *s*=32, *m* *c* *s* *s* *u* *b* *s* *t* *e* *p* *s*=64, *σ*_*max*_=2, *λ*=0.05, *t* *a* *r* *g* *e* *t* *A* *r* *e* *a* *s*=0.4, initial *d* *a* *r* *k*/*l* *i* *g* *h* *t* *r* *a* *t* *i* *o*=1/4

The temperature *T* functions as cellular motility factor since high *T* leads to frequent spin-copies, thus, an increase in the number of cellular bricks and an increase in cellular invasive radius. The impact of *T* on the overall run is highlighted in Fig. 4 (panel A). The default temperature is set to 10 degrees as suggested in [46, 75]. A comparison of our default settings with values, previously published by others, are summarized in Table 1.

**Table 1:** CPM parameter settings: comparison of presented default settings and values from literature [45, 46, 51, 75]

	Max X * Y	MCS, substeps	max *σ*	matrix density	T	J_*ECM*_	J_*light*_	J_*dark*_	J_*mixed*_	*λ*	A_*t*(*light*)_	A_*t*(*dark*)_	ratio_*light*/*dark*_
default settings	32 * 32	32, 64	2	0.8	10	16	15	2	11	0.05	0.4	0.4	1/4
GGH 1992	40 * 40	100, 1	2	1	10	16	14	2	11	1	0	0	1
GGH 1993	$\leq \sqrt {40*1000}*\sqrt {40*1000}$	16, max X * Y	1000	1	5	8–16	14	2	11	1	40	40	1
Ouchi 2003	128 * 128	1, 1	16	1	10	–/0	–5	–25	–3	10	64	64	1
Rubenstein 2008	500 * 500	400, Max X * Y	65	<0.1	0	0	2	2	9	1	40/2	50/2	1

The *parameter for area energy λ* represents a limiting factor to cell growth, also termed cellular elasticity *λ*. Panel B in Fig. 4 demonstrates the impact of *λ*. High *λ* values more strongly constrain cell growth while low *λ* leads to frequent spin-copies. The target area *A*_*t*_ is related to the lattice’s size parameters *x* and *y*, while the target area factors for light and dark cells can be adjusted.

The *energy interaction parameter J* is the basis to the overall Hamiltonian and spin-copy attempts. This so-called boundary energy coefficient determines cell growth as multiplicative degree of freedom [47]. Panels C to F in Fig. 4 illustrate the impact of low and high interaction values for different cells as light and dark cells and ECM on the overall simulation outcome and the underlying Hamiltonian and spin-copy attempts. The impact on the simulation by the parameter variables are presented within Fig. 4.

### Application example of cpm-cytoscape

We created a step-by-step tutorial on the presented tool using a tumor growth example based on parameters from a study on a brain cancer type modelled by CPM [51], available under https://github.com/davcem/cpm-cytoscape [74]. This example results in cellular growth of dark cells, representing tumor cells, showing a trend similar to Gompertz law. The simulated cancer cells thereby imitate 2D cultured glioma cells or likewise tumor-spheroids implanted in animals [51].

## Discussion

We present a web-based solution to allow for simple access to such a tumor growth visualization tool via Internet. By making use of the CPM implementation, we describe a potential use case for the cpm-cytoscape tool. The manipulable tool offers the advantage of adjustable settings for several input variables. By correlating various growth parameters we highlight the importance of heterogeneous cell interactions regarding its impact on tumor growth.

**Options to visualization:** There are many JS-based visualization libraries which can be used to foster the goals of visualization, namely to facilitate understanding and to gain novel insight, in our case into one of the many questions of biomedical research [76]. We make use of *Cytoscape.js* since it features user-friendly presentation of interaction data and supports several common browsers like Chrome, Firefox and Safari, while the first is the fastest one. It represents an open-source library on graph theory that was written in JS and developed for analysis and visualisation [73]. Thereby, layouts of the display area can be altered while graph elements can be accessed offering several possible operations including sorting and filtering as well as graph querying. These options can be exploited for future extensions to the tool. Moreover, Cytoscape.js [77] is regularly updated and supports directed as well as undirected, mixed or multi-graphs.

Furthermore, Cytoscape.js layouts can be easily changed by just specifying another graph layout for the layout parameter in the *cytoscapeRender* method. There are also alternative visualization libraries that can be used in the frontend [77–79]. Possible alternatives to Cytoscape’s layout algorithm would be using a bubble chart layout or even a three dimensional surface plot layout that can be created with another JS library such as D3.js.

Cytoscape.js offers different layout rendering options out of the box. We chose to use the grid layout that fits into traditional CPM visualization. In general, tumor growth kinetics and effects of cell growth can be visualized as line chart with the two dimensions of volume/size or cell number over time [80]. Therefore, we use the extension of simple line charts. Time series visualization may help users from the fact that time spans and iterations can dynamically be adjusted and are neither restricted by sensory constraints nor by experiment and animal costs.

**Lattice-based visualization of cells:** The lattice is organized in two dimensions, since 2D-modeling reduces the computational load just as well as visualization comprehensiveness. Still, in terms of numbers, the model could be manually transcribed and extended to a third dimension as the need arises.

In a figurative sense, the lattice represents tissue in the biological context. Cellular bricks are translated as textural compartments of a biological cell-layer. By way of example, the two-dimensional cellular grid can then be described as representative cross-section translated from the possible style of tissue slices. In a conceptional matter of speaking, cellular bricks represent variable compartmental states of a cell that can be translated to several criteria such as the impact of genes or likewise proteins, effects by modulators, inhibitors as well as promoters, or localized phenomena in general. The specific factors can be applied and extended in regard to the individual focus of research in a problem-directed manner.

**Initialization and lattice-site settings:** The variable number of lattice sites offers the possibility to adjust the computational workload according to the requirements of individual questions. In difference to general computational models, the Web-based implementation is attempted to be computed with low latency. Good rendering performance of computation results is needed to create dynamic output for smaller lattice sizes at once, as well as to enable animation for multiple computation steps at once. Still, some experiments concerning specific timing problems will have to be conducted using a high number of nodes. Thus, the variables can be be chosen in compliance with the requirements.

The random distribution, to a certain degree, simulates environmental behaviour and the random occurrence of mutations within cells. Spheroid models start from an initial mass of proliferative cells only. Still, in nature, mutated cells showing abnormal growth are intermixed with “normal” cells. Thereby, our tool allows to set various cell-types. Tumor cells are set to grow by means of proliferation and further invasion. The ECM can be set as background or individual cells to be equally or inhomogeneously in size and distribution [51]. For the future, we plan to implement extensions that will include additional initialization settings, such as the introduction of a dynamically configurable cell-type or another dimension. Further variations could include the option of spheroid models. Another elaborate feature could even offer pre-defined cellular mixtures corresponding to uploaded images from treated tissue-slices.

**The impact and translation for *MCS* and #***substeps***:** A MCS’s series of random copy attempts is equal to the total amount of cellular bricks. Graner and Glazier [45, 46] proposed *MCS* to be 16×*x*×*y* while *x*×*y*≈1000 and *x*=*y*≈<40 and did not make use of defining substeps. They suggested this setting for observing gradual movement behaviour. Later works define one *MCS* to consist of as many index-change attempts as the number of pixels in the lattice *x*×*y*. If the setting for *MCS*×*#substeps* is lower than *x*×*y*, then unintended results are observed.

The time, by means of *MCS* steps, is an abstraction and relates to tumor specifics. The various kinds of tumor cells proliferate and divide more frequently than normal cells, depending on the localities and their differentiation status. Thereby, tumors can be classified by their spatial occurrence, and further, be characterized by their temporal growth dynamics. For each case, MCS steps can be translated to either hours, days or years. Future extensions to our tool will include pre-defined initialization settings of growth rates and time units corresponding to exemplary tumor types.

**Temperature ***T***:** In general, temperature affects movement, and in our case, cell growth. In more detail, *T* functions like a cellular motility factor since high *T* will lead to frequent division of cells, thus, an increase in the number of cellular bricks and an increase in cellular invasive radius as shown in Fig. 4 (Panel A). If the interaction energy, represented by the several J parameters, is much greater than T, cells will shed into loose bricks at the boundaries. If T is too large, relative to J, boundaries will become stiff. Low temperatures inhibit proliferation. Subzero temperatures stop changing spin values and therefore kinetics and growth. At very low subzero temperatures, any biological activity is effectively stopped but cells could also take damage through freezing, that could be taken into account as additional factor in future studies.

**The energy interaction parameter ***J***:** The range of the individual interaction energies is defined by the original cell-types as well as the manifested mutations responsible for the excessive proliferation by tumor cells. Thereby, these factors correlate with the class of tumor and it’s tissue-residency. Individual cells exhibit heterogeneous tendencies towards growth correlating to tumor aggressivity, thus, interaction energies can vary over time. This phenomenon can be manually emulated by adjusting the individual interaction parameters after a specified number of MCS. Future extensions could include this adjustment as an automatic option in correlation to underlying relations of further variables.

In our case, default parameters of cpm-cytoscape implicate low values within the first term for the Hamiltonian computation, consisting of the interaction parameters *J*, in comparison to the second term, factoring values of area calculation such as *λ*, *a* and *A*_*t*_ (see details to Eq. 4). As can be seen in Fig. 4 (panel C) a change in *J*_*mix*_, the interaction energy between different cells, impacts growth of dark cells considerably. However, there are no significant differences if the *J* parameter of dark or light cells is changed selectively (panel F). Changes of *J*_*ECM*_, the interaction energy between parts of ECM, result in similar insignificance, though high values can lead to sudden changes in the ratio between dark and light cells through dark cells migrating to and taking over former ECM space (panel E). Rather high values are needed to manipulate ratios. Figure 4 (panel D) demonstrates three cases of combined changes in the interaction parameters *J*_*dark*_, the interaction energy of dark cells, in comparison to the interaction energy of light cells *J*_*light*_, as well as *J*_*mix*_ and *J*_*ecm*_. The ratio between dark and light cells is only slightly decreased upon an 100-fold increase of *J*_*dark*_. However, the number of dark cells over light cells is completely reduced upon increasing *J*_*mix*_ and *J*_*ecm*_. At the same time, the relation between *J*_*dark*_ and *J*_*light*_ plays a minor role in determining the probability of spin-copy attempts rather to their measure in proportion to *J*_*mix*_ and *J*_*ecm*_. This fact can be translated to the biological importance of heterogeneous interactions between cells and their environment. Further refinement will include the integration of additional parameters such as *J*_*dark*−*ecm*_, *J*_*light*−*ecm*_ or other *J*_*diff*_ as well as the search for suitable realistic values to relate to different cell-types, a factor to be taken into account in future studies.

**The target area and the parameter for area energy ***λ***:** The factor *λ* is considered a constraint, in our case, for limiting cell growth. The so-called cellular elasticity *λ* attaches the value of area calculation within the Hamiltonian computation. Differences between current and target area will likely have more effect on spin-copy attempts if *λ* is high. If *λ* becomes too high relative to the residual calculation parameters, any spin-copy attempt should be refused. This is true as long as the cellular area is different from the target area. The quadratic function does not distinguish whether the cellular area is larger or smaller than the target area. In terms of cell size, cellular elasticity will play a major role for rigid cells which tend to stay within the range of their target volume. Cell growth and division are correlated so that cells of unequal size will divide at a given speed and even out to a mean cell size. This is true only, if cell growth rate is constant. An abnormal increase in cell size is possible under the influence of excessive discharge of growth hormones or similar pathological circumstances such as hypertrophy. Other cases of instant changes in cell size include the natural processes of cellular differentiation and enlargement or shrinkage according to the metabolic state.

Generally, various cell-types are differently sized. Some cancers are known to manifest giant cells. Even normal cells exhibit different dimensions according to their origin. Cell diameters range from 1*μm* to 1*mm* and more, for instance nerve cells can reach a length over 1*m* [81]. Furthermore, cell-sizes vary within one cell-type. Still, cells have medial sizes specific to their type. This constraint is thereby necessary to limit cellular growth to an underlying biological scale.

For future matters, the discrete view of cellular area can have a completely different meaning. Cellular bricks resemble conceptional factors that occur or are replaced, distributed or accumulated within individual cells. These factors will be assigned by the researcher depending on a given task and scope of work.

**The ECM** occupies space which is not attributed to cellular clusters. Its energy area is initially suppressed, but if reprogrammed to a positive number within the source code, the ECM will grow and spread like light and dark cells. This could simulate gap-filling after cell-death and be the case of radiation procedures, cellular starvation or exposure effects of chemicals. This variation will be of importance in future studies introducing multiple affectors of cell growth by integration of biomedical databases, including drug, protein and genetic information related to tumor growth.

**The matrix density** was introduced as factor for simulating various cell densities within the area of interest. For instance, tissue slices could show distinct cellular colonization in locally fragmented patterns. Moreover, different cell-types as well as organelles can exhibit various densities. In general, varying cell densities can be attributed to the water content relative to the mass of proteins, nucleotides, carbohydrates or lipids within and around the cells.

Cell density often resembles the proliferative state of cells controlling protein expression. Consequently, the change in matrix density can be used for future studies focusing its effect on tumor growth, dormancy or metastasis. Further, matrix density can be interpreted in a more formalized manner, such as the variable abundance and occurrence of discrete factors within cellular regions.

**The role of fostering in silico modeling:** There is a trend towards computational simulations of biological processes making use of different mathematical models [82]. In particular simulation-based experiments in the field of bioinformatical cancer research can save resources in terms of time and costs. Collaboration between experimentalists and modelers has to be promoted and extended. This fact is most interesting for fostering cooperation of researchers from the interdisciplinary fields of computer science, mathematics, human-computer interaction, life sciences and biomedicine [83].

The tool represents a basic instrument to supporting biomedical researches and a preliminary step towards supporting clinical scientists. Until now, the tool has not been evaluated by clinicians. Future plans are to conduct further iterative testing and verification and to experiment with machine learning approaches [84].

## Conclusion

Recent advances in Computational Biology show high potential to deepen the understanding of origin and progression of cancer. Our general aim is to enrich cancer research by providing a tool that will make Computational Biology applicable to both researchers and clinicians. We focus on the fundamental pathological processes of cancer which are represented by tumor growth. Since abnormal cell growth involves chaotic, heterogeneous and highly differentiated structures, we chose to investigate cellular growth on the single-cell level. By refining model parameters of the cellular potts model, we highlight the impact of heterogeneous interceullular interactions on tumor growth.

Herein, we describe the implementation of the CPM for the purpose of simulation and visual analysis of tumor growth and provide its sources on github. We chose the lattice-based visualization style as primary approach to present and display tumor growth for research purposes. The graph computation allows for multiple different visualization approaches. The user interface is highly adjustable and its implementation is designed to be extended. The possibilities and accessibility of our simulation and visualization approach might ultimately promote researchers and practitioners to progressing the field of tumor research towards personalized medicine.

Our approach offers several potential future applications of studying tumor dynamics. First, we plan to implement more simplistic models in order to offer fast computations and visualizations. Secondly, we plan to integrate various profiles into the tool, to offer exemplary simulations on different types of tumors [74]. Next to iterative testing, profiles lead to the task of verification. Furthermore, the implementation of additional dynamic parameters may enhance the simulation’s possibilities. Multiple optional features to modeling as well as visualization styles will provide preferential outcomes in regard to detailed information or fast overview performance. Another interesting step towards supporting researchers and clinicians is providing image loading and size detection of regions of interests as input parameter for the simulation. Future integrations will include biomolecular networks such as drug-protein impact or genetic alteration patterns. Harnessing tumor growth data and related gene data as well as providing an open source database for tumor growth related data [85] are big steps forward to supporting science collaborations and clinical applications, and finally help contributing to fight cancer.

We believe that our approach is a motivator for fostering in silico modeling towards 3R and a better understanding of tumor dynamics.

## Abbreviations

CPM, cellular Potts model; ECM, extracellular matrix; GGH, Glazier-Graner-Hogeweg; JS, javascript; MCS, Monte Carlo step
